# Supplementary material for: MoaE Is Involved in Response to Oxidative Stress in Deinococcus radiodurans
Source: Int J Mol Sci. 2023 Jan 26;24(3):2441. doi: 10.3390/ijms24032441 (PMC9916421; doi:10.3390/ijms24032441)
Supplement: Supplementary file 1 [file ijms-24-02441-s001.zip › ijms-2106517-supplementary.pdf]

Supplementary Materials

MoaE is involved in response to oxidative stress in *Deinococcus radiodurans*

Supplementary Table S1. Data collection, phasing and refinement statistics of DrMoaE crystal diffraction.

	**DrMoaE**
**Data collection**	
Space group	$C222_1$
Cell dimensions	
a, *b*, *c* (Å)	62.02
	94.95
	101.11
Wavelength (Å)	0.9792
Resolution (Å)	30.0-2.00
R_{sym} (%)	5.7 (56.0)
$I/\sigma I$	16.5 (2.9)
Completeness (%)	99.9 (100.0)
Redundancy	6.4 (6.7)
**Refinement**	
Resolution (Å)	30.0-2.00
No. reflections	20510
R_{work}/R_{free}	19.4/23.6
No. atoms	
Protein	2121
Ligand	10
Solvent	77
B-factors	
Protein	51.8
Ligand	56.6
Solvent	45.9
R.m.s deviations	
Bond lengths (Å)	0.005
Bond angles (º)	0.74

*Highest resolution shell is shown in parenthesis.

Supplementary Table S2. Strains and plasmids used in this experiment.

**Strains**	**Relevant characteristics**	**Reference or source**
D. radiodurans		
DraR1 wt	Wild-type strain ATCC13939	Laboratory stock
ΔdrmoaE	R1 but *drmoaE::str*	This study
ΔdrmoaE_Cwt	*ΔdrmoaE* but pRADK*::drmoaE*	This study
ΔdrmoaE_R110A	*ΔdrmoaE* but pRADK*::drmoaER110A*	This study
Escherichia coli		
DH5α	Cloning strain	TransGen
BL21 (DE3)	Expression strain	TransGen
**plasmids**		
pET28a	T7 promoter, T7 terminator, Kana, 6×His-tag coding sequence	Novagen
pET28aMoaE	pET28a containing wild type *moaE* gene	This study
pET28aMoaE R110A	pET28a containing *moaE* site mutation R110A gene	This study
PRADK	*E. coli-D. radiodurans* shuttle vector	Laboratory stock
pRADK-*drmoaE*	pRADK*::drmoaE*	This study
pRADK-*drmoaER110A*	pRADK*::drmoaER110A*	This study

Supplementary Table S3. Primers used in this experiment.

Primers	Sequence (5′-3′)
**Expression of proteins**	
moaE-F (NdeI)	TTTTTTTCATATGATGGCCCCCGAGGACGAG
moaE-R (BamHI)	TTTGGATCCTCACAGCGTGTCGTGGCC
moaER110A-F	GCGCCGGGCGGGCGTGGGGGCTTG
moaER110A-R	CAAGCCCCCACGCCCGCCCGGCGC
**Construction and complement of mutant stains**	
moaE-P1	GTCGCTGGCCCCAAATTTCT
moaE-P2 (HindIII)	TTTAAGCTTACCGCCCGCCACCGG
moaE-P3 (BamHI)	TTTGGATCCGACTTCGGTAAAAACGCTGTCCTGT
moaE-P4	GTTTTACCCGCCGCACGC
moaE-P5	ACGCGGACACCCACTGCC
moaE-P6	GGTGGGGGCTTGCGACAC
ΔmoaE_Cwt-F	TTTTTTTCATATGATGGCCCCCGAGGACGAG
ΔmoaE_Cwt-R	TTTGGATCCTCACAGCGTGTCGTGGCC
**Real time PCR primers**	
moaE-RTF	ACGCGGACACCCACTGCC
moaE-RTR	GGTGGGGGCTTGCGACAC
drxdhB-RTF	AGGGCGAGTTCGAGTTCGGC
drxdhB-RTR	CTCAGAAVGGCTCCAAGCGC
dr_0397-RT-F	TTGATTGAGTGGCCCCGAGAT
dr_0397-RT-R	GTAGTGGACCGTCTTGGCACAG
dr_1343-RT-F	GAAAGTAGGCATCAACGGCTTT
dr_1343-RT-R	TCCACGGTGCCGTCAAAG

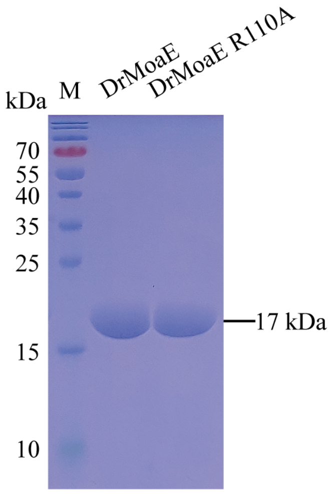

Supplementary Figure S1. The SDS-PAGE (15%) analysis of DrMoaE and DrMoaER110A.
